# Notes and News

**Published:** 1904-02-13

**Authors:** 


### NOTES AND NEWS.

THE Prince and Princess of Wales have promised to attend the fête at Claridge's Hotel, on behalf of the League of Mercy, to be held on the 15th and 16th instant.

THE late Mr. John Snelgrove, of the firm of Marshall and Snelgrove, bequeathed £500 each to the Middlesex Hospital and the London Fever Hospital.

THE annual festival ball in aid of the Italian Hospital, Queen Square, W.C., will be held at the galleries of the Royal Institute of Painters in Water Colours on Monday, May 9th, under the patronage of their Royal Highnesses, the Prince and Princess of Wales, the Duke and Duchess of Aosta, the Duke and Duchess of Connaught, Prince and Princess Christian of Schleswig-Holstein, Princess Louise, Princess Henry of Battenberg, his Excellency the Italian Ambassador and Madame Pansa, and the staff of the Italian Embassy, the Italian Consul-General, the Duchesses of Devonshire, Marlborough, and Buccleuch, the Marchionesses of Lansdowne, Londonderry, Salisbury, and Tweeddale, Countess Roberts, the Ladies Edmund Talbot and Mary Howard, and the Lord Mayor and Lady Mayoress.

THE Radcliffe Infirmary, Oxford, is in considerable financial difficulty. At the last quarterly court this was carefully considered. It was pointed out by the chairman that it would be necessary either to close a ward or secure increased subscriptions. Although the infirmary is not ministering to an appreciably larger number of in-patients than during several decades past, the modern standard of efficiency has demanded a greatly increased expenditure. The infirmary has now its Finsen light and Roentgen ray departments, and possesses a resident medical officer, and in every direction there is evidence of an endeavour to render the ministrations of the hospital complete and effective. Under these circumstances, it would be a matter of deep regret and a lasting reproach to Oxford, its University, city and county, if the only means of carrying on its labours without incurring a ruinous liability, were to reduce the volume of its good work; we hope that the alternate plan proposed of an organised attempt to secure increased support will render such a step quite unnecessary. The division of Oxford and its county into localities in which ladies will undertake each her own section, in which to arouse interest and secure contributions, seems a very promising proposal, and the public meeting in the Town Hall should further awaken attention and produce a good result.

A NEW block of infirmary buildings has been added to the Swansea Workhouse. It contains a women's receiving ward, vagrant wards, general wards, lying-in ward, nurses' quarters, surgery, offices, and a disinfecting department. The accommodation is for 71 patients, and the cost is estimated at about £17,000. The architect is Mr. H. Wills, London.

THE question of introducing lady visitors on the management of the Dumfries and Galloway Royal Infirmary, was the subject of an interesting discussion at the last annual meeting of Governors. The innovation was proposed under the plea that the experience of ladies in household management could be usefully placed at the disposal of the Directorate, by the creation of a committee of lady visitors to report to the Directorate, and the number of 12 to 15 was specified in the proposal. The scheme was opposed on the ground that the admission of a number of irresponsible ladies, bent on discovering defects, might seriously hamper and disturb the work of the institution; the large number of ladies proposed was also objected to. The matter ended by the mover withdrawing the motion, leaving it to the further consideration of the directors, and to be brought up at an adjourned meeting of the Governors.

THE late Mr. Edmond Dresden, of Curzon Street, Mayfair, has bequeathed his pictures to the Children's Hospital at Great Ormond Street, to be hung in the wards, and a sum of £25,000 for the maintenance in perpetuity of beds, to be called the "Dresden Cots," which are to be contained, if possible, in one ward, such ward to be called the "Dresden Ward;" £5,000 each to the Middlesex Hospital, St. Mary's Hospital, Paddington, the Royal Free Hospital, Gray's Inn Road, the Hospital for Consumption at Brompton, and Queen Charlotte's Lying-in Hospital; but these legacies, and all other legacies bequeathed by him, are to be for the benefit of the patients, and no part thereof is to be expended for building, furnishing, or decorating. Mr. Dresden left the residue of his property, after sundry personal bequests, as to one-fifth each for the Middlesex Hospital, St. Mary's Hospital, the Brompton Hospital for Consumption, the Royal Free Hospital, and Queen Charlotte's Lying-in Hospital, but on condition that such legacy is in each case to be held in trust to create a fund to be called the "Dresden Assistance Fund," and the income thereof is to be applied for the benefit of needy and deserving in-patients of the hospital on their being discharged. The testator left his share of the reversionary interest in his sister's settlement in equal shares to the Middlesex Hospital, the London Hospital, and the Cancer Hospital at Brompton, for the purpose of providing beds to be called the "Dresden Beds," and for the benefit of the patients, but in no case to be applied for the purpose of building or furnishing. The total amount available for Mr. Dresden's charitable bequests seems likely to exceed £250,000.

